# GC-IMS combined with sensory and chemical analyses to reveal flavor and taste differences of antarctic krill paste under different processing conditions

**DOI:** 10.3389/fnut.2026.1805406

**Published:** 2026-04-13

**Authors:** Peng-Fei Jiang, Shi-Meng Sun, Jing Li, Yang Liu, Jing-Ru Chen, Jia-Bo Huang

**Affiliations:** SKL of Marine Food Processing and Safety Control, National Engineering Research Center of Seafood, School of Food Science and Technology, Dalian Polytechnic University, Dalian, China

**Keywords:** Antarctic krill paste, electronic nose, GC-IMS, maturation method, sensory properties

## Abstract

Existing research on shrimp paste has focused primarily on fermentation optimization and microbial analysis, while systematic studies on the impact of different processing methods on its flavor are notably scarce. This study investigated the flavor characteristics of Antarctic krill paste processed using different cooking methods (stir-frying versus steaming), as well as the effects of salt concentration variation and aged shrimp paste addition on overall quality. Gas chromatography-ion mobility spectrometry (GC-IMS) analysis identified 42 volatile compounds, with aldehydes and ketones present at significantly higher levels than other compounds. Compared to steamed krill paste samples, stir-fried samples exhibited higher levels of free amino acids contributing to umami and sweetness, and elicited stronger umami and saltiness responses in electronic tongue testing. Electronic nose and principal component analysis results indicated clear differentiation between stir-fried and steamed krill paste based on volatile profiles, with stir-fried samples exhibiting greater flavor variability. Sensory evaluation further confirmed that the 25% salt concentration stir-fried krill paste (CK25) achieved the highest rating due to its richer flavor and more harmonious texture. These findings indicate that the stir-frying process with an optimal salt concentration (25%) is the preferred method for enhancing the flavor and textural quality of Antarctic krill paste, providing a theoretical basis for optimizing its industrial production process.

## 1 Introduction

Antarctic krill (*Euphausia superba*) is a species with significant resource potential within the Antarctic ecosystem. Its rich nutritional profile makes it an ideal raw material for food development (1). Protein, as the primary nutrient in Antarctic krill, is not only abundant but also exhibits a balanced amino acid composition, containing all nine essential amino acids required by the human body. It is easily digestible and absorbable (2).

Additionally, the EPA and DHA abundant in Antarctic krill play a significant role in human cardiovascular health and brain development. Antioxidant substances like astaxanthin possess physiological functions such as anti-inflammatory and antioxidant properties. These characteristics give Antarctic krill broad application prospects in the food industry (3). A traditional fermented seafood product, shrimp paste enjoys great favor among consumers for its exceptional flavor attributes and ample nutritional constituents (4). With its distinctive savory flavor profile, Antarctic krill serves as an exceptional raw material for developing specialty shrimp paste products. During food processing, maturation represents a critical stage that significantly impacts the final product quality. Different maturation methods alter heat transfer efficiency and reaction environments, triggering a series of biochemical reactions such as protein degradation, fat oxidation, and the Maillard reaction. These processes subsequently influence the food’s flavor, taste, and nutritional composition (5). Steaming and stir-frying are two commonly used methods of cooking in food processing. The former employs steam as the heat transfer medium, providing gentle and uniform heating that effectively preserves nutrients. The latter uses oil as the heat transfer medium, offering rapid heating that readily develops rich flavor compounds but may result in the loss of certain nutrients.

Currently, research on shrimp paste primarily focuses on optimizing fermentation processes and analyzing microbial community structures, while systematic studies examining the effects of different maturation methods on its flavor and taste profile remain relatively scarce. Flavor and taste serve as core indicators for evaluating shrimp paste quality (6). Volatile flavor compounds determine its odor characteristics, while taste-related substances such as free amino acids influence its sensory experience. As a rapid odor detection instrument, the electronic nose can sensitively distinguish different types of flavor compounds. GC-IMS integrates the strong separation performance of gas chromatography with the high sensitivity of ion mobility spectrometry, thereby facilitating rapid qualitative and quantitative analysis of volatile compounds. It provides crucial data to support shelf-life information (7), variety quality (8), and processing technology optimization (9). Electronic tongues simulate the human taste system to objectively evaluate food flavor characteristics; free amino acid analysis identifies key chemical components influencing food taste.

This study selected Antarctic krill paste samples with varying salt concentrations (20%, 25%) and aged shrimp paste additions (5%) as research subjects. These samples underwent maturation treatment via two distinct methods: steaming and stir-frying. The study hypothesizes that optimizing salt concentration and selecting the optimal aging method can more effectively enhance the flavor, texture, and sensory quality of shrimp paste, and that these differences can be detected using gas chromatography–mass spectrometry (GC-IMS), electronic nose and sensory evaluation methods. This systematic investigation aims to clarify the patterns of change in key flavor and taste compounds, providing theoretical support and technical references for optimizing Antarctic krill paste maturation processes and enhancing product quality.

## 2 Materials and methods

### 2.1 Sample preparation

The Antarctic krill used in the experiment were provided by Liaoyu Group Co., Ltd. and transported to the laboratory in an insulated box equipped with ice packs to ensure they had not thawed upon arrival. To remove moisture, frozen krill meat was thawed at 4°C, then divided into equal portions and pressed using a stainless steel manual press (0.3 MPa, 2 min) to expel excess moisture. The samples were then reweighed to ensure uniform moisture removal across all batches, thereby eliminating experimental variations caused by uneven moisture levels. The moisture-squeezed Antarctic krill was then mixed with fermented shrimp paste in equal proportions. Add an appropriate amount of soybean oil, maintaining a mass ratio of krill meat: shrimp paste: oil = 10:1:5. Four sample formulations were prepared: 20% salt content (K20), 25% salt content (K25), 20% salt content + 5% aged shrimp paste (CA), and 25% salt content + 5% aged shrimp paste (CC). Each formulation was stirred for 2 min to ensure uniform distribution of ingredients. Fifty grams of sample were weighed and portioned into sterile containers for cooking trials. All treatment groups maintained consistent batch sizes to standardize heat transfer kinetics.

Each formulation underwent two distinct cooking methods: steaming (Z) and stir-frying (C). Steaming was performed using a multifunctional steam oven (SCC WE10, Shanghai Yuena Industrial Co., Ltd., China) at 100°C for 10 min. The container used in the steam oven was a heat-resistant borosilicate glass jar (50 mL capacity) fitted with a snug-fitting lid to ensure vapor permeability while preventing water vapor condensation from dripping into the sample. The stir-frying process was conducted using a multifunctional cooking machine (TM6-1, Ningbo Xinhua Educational Equipment Co., Ltd., China) at 100°C for 10 min. This yielded eight shrimp paste samples: CK20, CK25, CCA, CCC, ZK20, ZK25, ZCA, ZCC.

All sample processing, pretreatment, and instrumental testing operations were conducted under strictly controlled environmental conditions: sample preparation and low-temperature preprocessing (4°C) for free amino acid extraction; instrumental detection (22°C) for GC-IMS, electronic nose and amino acid analysis. All operations were performed under light-shielding or neutral light conditions to avoid light-induced quality changes, and all samples were equilibrated to the corresponding test temperature before analysis to ensure experimental consistency.

### 2.2 GC-IMS analysis

Following the method described in Huang et al. (10), accurately weigh 5.0 g of matured shrimp paste sample and place it in a 20 mL headspace vial. Prior to injection and analytical procedures, the samples were subjected to incubation at 60°C for 15 min, and all analyses were performed in triplicate for each sample. Analysis was performed using a FlavourSpec^®^ flavor analyzer (Discovery HR-1, G.A.S. GmbH, Germany) with an MXT-5 column (30 m × 0.53 mm × 1 μm). The headspace injection conditions are as follows: Incubation temperature: 80°C Injection volume: 500 μL Non-split injection Incubation speed: 500 r/min Injection needle temperature: 85°C.

GC Conditions: Column temperature: 60°C; Carrier gas: High-purity nitrogen (purity ≥ 99.999%); Programed flow ramp: Initial flow rate 2.0 mL/min held for 2 min, linearly increased to 10.0 mL/min over 8 min, then linearly increased to 100.0 mL/min over 10 min. Chromatography runtime: 20 min for sample; Inlet temperature: 80°C. IMS Conditions: Ion Source: Tritium source (^3^H); Traveling Tube Length: 53 mm; Electric Field Strength: 500 V/cm; Traveling Tube Temperature: 45°C; Drift Gas: High-purity nitrogen (purity ≥ 99.999%); Flow Rate: 150 mL/min; Positive Ion Mode. Volatile components were qualitatively analyzed by matching the retention time and drift time of detected signals with the NIST and IMS databases integrated into the FlavourSpec^®^ analyzer; semi-quantitative analysis was conducted based on the relative peak area of each compound, with peak areas normalized against the reaction ion peak (RIP) to minimize instrumental variability. Statistical analysis of the volatile compound data was performed using a multi-range test (*p* < 0.05) to identify significant differences in compound concentrations among samples.

### 2.3 Electronic nose analysis

An accurately weighed 2 g portion of shrimp paste sample was taken. The sample was incubated in a constant-temperature water bath at 60°C for 30 min, and then analyzed using an electronic nose (PEN3, AIRSENSE Analytics GmbH, Germany). The method described by Kong et al. (11) was adopted with slight modifications, and the parameters of the electronic nose were set as follows: flushing time 20 s, sampling time 70 s, sampling interval 1 s. Each sample was subjected to three parallel measurements, and the data from 50 to 52 s were selected for analysis. The PEN3 electronic nose incorporated 10 sensors, each of which was sensitive to different types of volatile compounds. The performance of the sensors was detailed in Supplementary Table 1.

### 2.4 Electronic tongue analysis

Mix matured shrimp paste with deionized water at a ratio of 1:10, then homogenize using a homogenizer. Centrifuge the homogenate at 8,000 rpm for 10 min at 4°C (12). After centrifugation, the supernatant was collected and filtered through a 0.22μm membrane filter for subsequent sample analysis. An electronic tongue (TS-5000Z, Insent Co., Ltd., Japan) was used for measurement. Each sample was measured four times, and the last three results were used for analysis.

### 2.5 Free amino acid analysis

Following the method described in Wang et al. (13) with minor modifications. Accurately weigh 1 g of shrimp paste sample, dissolve in 0.02 mol/L HCl solution, and dilute to 25 mL. After 20 min of ultrasonic extraction, centrifugation was performed at 6,000 rpm for 10 min. The resulting supernatant was filtered with a 0.22 μm membrane.

Free amino acid quantification was conducted on a HITACHI L-8900 amino acid analyzer (Hitachi High-Technologies Corporation, Tokyo, Japan) with the experimental parameters as follows: a constant flow rate of 20 mL/h was applied for the buffer solution and 10 mL/h for the reaction solution. A sodium-form cation exchange resin column with specifications of 200 mm × 4.6 mm (length × inner diameter) was employed for chromatographic separation of amino acids. Dual-wavelength ultraviolet (UV) detection was implemented at 570 and 440 nm, respectively. A stepwise temperature program was adopted for the separation column, with the temperature elevated progressively from 55 to 65°C and further to 77°C; in the meantime, the temperature of the reaction tank was kept constant at 138°C during the entire analytical process. The inlet and injection volumes were both set at 50 μL for all assays. Amino Acid Standard (AAS18) was sourced from Sigma-Aldrich (St. Louis, MO, United States).

### 2.6 Sensory evaluation

A panel of 14 food science students (7 males, 7 females) with sensory evaluation training was selected (14). Samples were scored across four attributes: color, texture, aroma and taste, based on the sensory evaluation criteria in Table 1. The panel members completed targeted training, which included theoretical instruction on the sensory attributes and scoring criteria for shrimp paste, as well as practical blind tasting calibration sessions to ensure consistent evaluation standards. The formal evaluation was conducted in a standard sensory laboratory (25°C, odor-free, well-lit).

**TABLE 1:** Sensory evaluation criteria of shrimp paste.

Evaluation index	Scoring criteria
Color	Clear and bright oil color with distinct glossiness (7–10)
	Moderate oil color with slight glossiness (3–6)
	Turbid oil color with poor glossiness (0–2)
Texture	Moderate viscosity with homogeneous texture (8–10)
	Average viscosity with relatively homogeneous texture (6–8)
	Slightly poor viscosity with minor stratification (3–6)
	Poor viscosity with distinct stratification (0–3)
Aroma	Distinct umami flavor characteristic of shrimp paste (7–10)
	Weak shrimp paste umami flavor masked by oil odor (3–6)
	No shrimp paste umami flavor, with strong fishy odor (0–2)
Taste	Moderate salinity, with fresh and sweet shrimp flavor (8–10)
	Mild umami, balanced salinity and sweetness, free of fishy odor (6–8)
	Mild umami with a slightly salty taste (3–6)
	No umami, with prominent salinity (0–2)

Ethical approval was not required for the sensory evaluation in this study, as it only involved the tasting of a common processed seafood product with no invasive operations, biological sample collection, or potential health risks to participants, in line with the research ethics guidelines of Dalian Polytechnic University.

### 2.7 Statistical analysis

Data statistical analysis was performed using SPSS 26.0 software, with single-factor analysis of variance (ANOVA) employed for significance testing (*P* < 0.05). Origin 2021 software was utilized to generate radar charts, PCA plots, and other graphical representations. GC-IMS data were qualitatively and quantitatively analyzed using LAV (Laboratory Analytical Viewer) software Version 4.5.0 (G.A.S., Dortmund, Germany).

## 3 Results and discussion

### 3.1 Detection of volatile compounds in shrimp paste using GC-IMS

#### 3.1.1 GC-IMS spectra of shrimp paste using different processes

**FIGURE 1:**
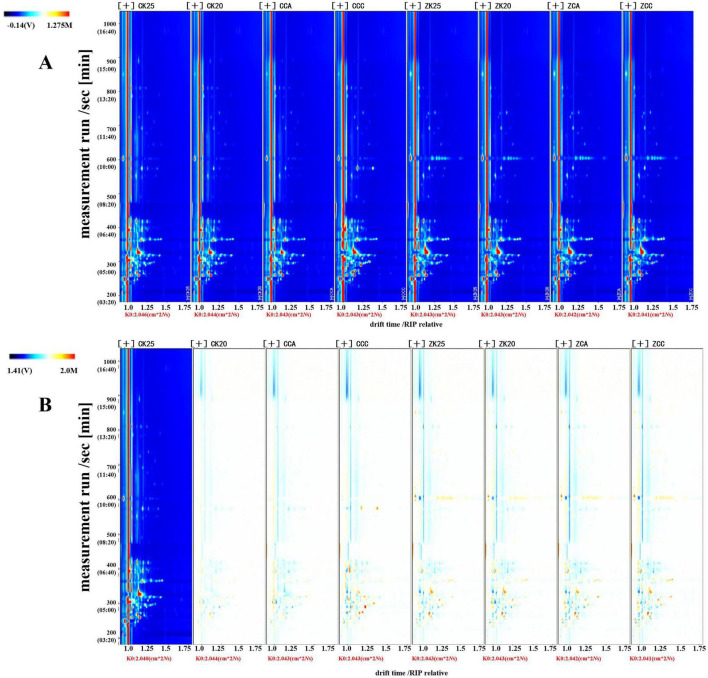
GC-IMS two-dimensional spectrum of volatile components in shrimp paste **(A)** and GC-IMS differential spectrum of volatile components **(B)**.

As shown in Figure 1A, the GC-IMS spectrum. The red vertical lines correspond to normalized reaction ion peaks (RIP peaks), while each discrete spot represents a single volatile organic compound (VOC). The color intensity of each spot directly reflects the concentration of its corresponding compound: deep red indicates high concentration, while white signifies low concentration. Color intensity increases with rising VOC abundance (15). Most VOCs are concentrated in the range of 200–600 s for retention time and 1.0–1.5 ms for drift time, a phenomenon that indicates the dominant VOCs in this area show strong reactivity. Furthermore, the consistent spatial distribution of spots across all eight samples indicates significant similarity in the overall volatile organic compound profiles between stir-fried and steamed shrimp paste groups. However, subtle variations in spot hue intensity suggest that processing methods modulate the concentration of specific flavor compounds.

To compare flavor differences, a differential comparison model was employed, with the stir-fried shrimp paste sample (CK25: 25% salt content, no aged shrimp paste added) serving as the reference group. Volatile component profiles for the remaining samples were obtained through differential analysis against the CK25 profile. In this model, white areas indicate no difference from the reference group; red regions signify higher compound content than the control, while blue regions indicate the opposite (16). As shown, within the retention time range of 300–600 s, the steamed group exhibited stronger red signals than the stir-fried group, indicating that steaming promotes the accumulation of specific flavor compounds. For samples supplemented with aged shrimp paste, CCC exhibited stronger red signals than CCA. This suggests that the 25% salt concentration combined with aged shrimp paste addition is more conducive to volatile compound formation, likely due to this salt level optimizing microbial metabolic activity during processing. Furthermore, under identical salt concentration and aged shrimp paste combinations, the steamed group consistently exhibited stronger red signals than the stir-fried group. This indicates that steaming enhances the formation of aromatic compounds, likely due to its gentle heat transfer preserving enzymatic and microbial activity (17). Although significant differences in volatile flavor compounds were observed between processing methods and between salt concentration and aged shrimp paste treatment groups, further analysis of compound-specific fingerprinting is required to clarify changes in individual compounds.

#### 3.1.2 Qualitative analysis of volatile components

**FIGURE 2:**
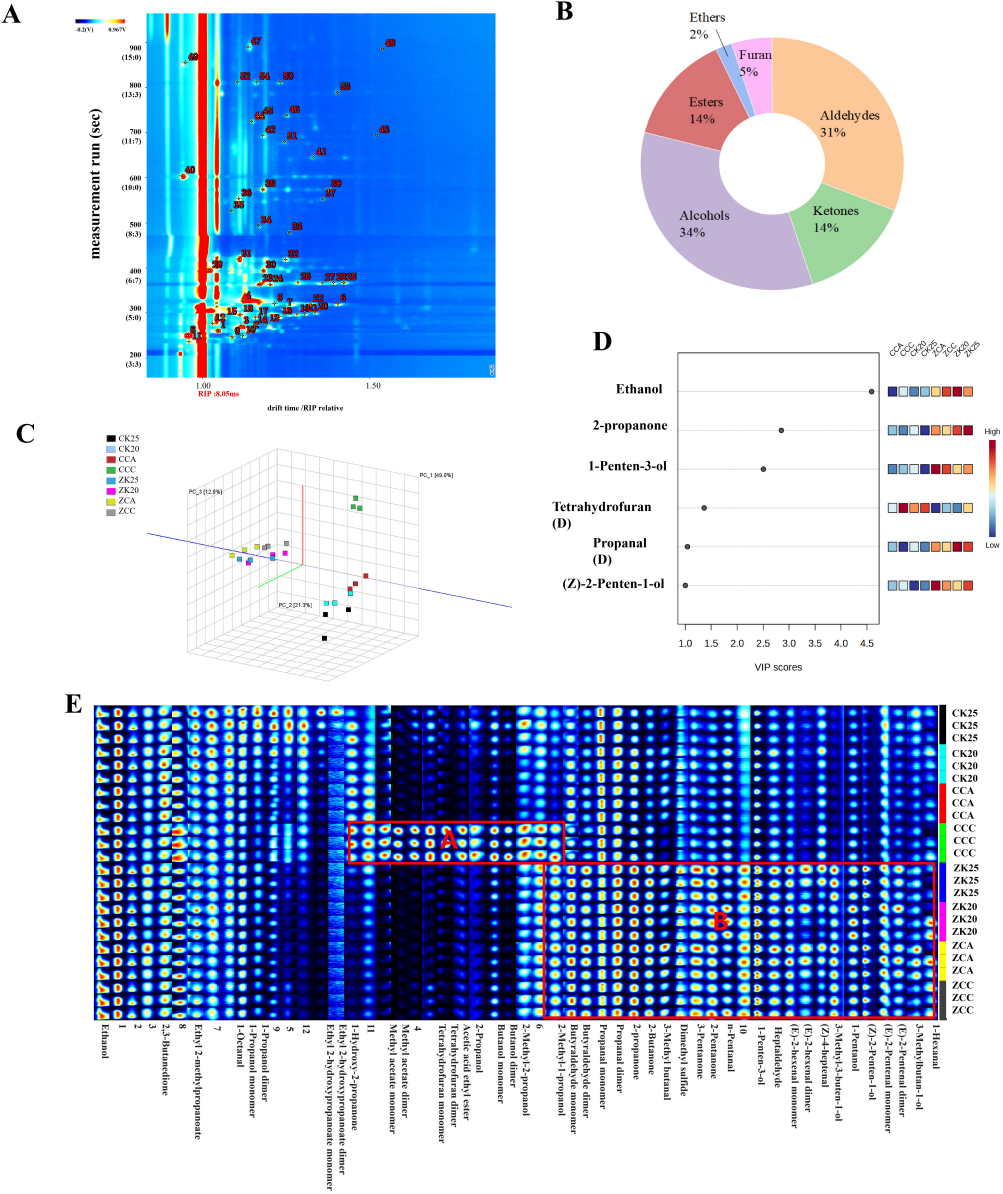
Characteristics of volatile flavor compounds in shrimp paste under different processing methods and qualitative analysis of volatile substances **(A)**; number and percentage of volatile compounds **(B)**; PCA score of volatile components of shrimp paste cooked in different ways **(C)**; volatile flavor compounds with VIP value > 1 **(D)** and fingerprints of volatile flavor compounds in shrimp paste cooked in different ways **(E)**.

Qualitative analysis findings for volatile flavor components are displayed in Figure 2A. Table 2 provides the correspondence between each number and a specific volatile flavor compound, in which “M” and “D” stand for the monomer and dimer forms of the compound, respectively (18). By aligning the retention times of these volatile flavor compounds with entries in the IMS database, GC-IMS technology was utilized for the analysis of volatile components in shrimp paste samples. A total of 54 volatile compounds were detected, with 42 successfully identified. These included 13 aldehydes, 6 ketones, 14 alcohols, 6 esters, dimethyl sulfide, and tetrahydrofuran.

**TABLE 2:** Volatile flavor compounds in cooked shrimp paste by different methods.

Count	Compound	Oder type	CAS	Formula	MW	RI	Rt[sec]	Dt (a.u.)
1	Propanal (M)	Pungent, green grassy	C123386	C3H6O	58.1	795.4	260.412	1.04612
2	Propanal (D)	Pungent, green grassy	C123386	C3H6O	58.1	794.4	259.981	1.14648
3	2-propanone	Fresh, apple, pear	C67641	C3H6O	58.1	818.2	270.332	1.11683
4	Ethanol	Aromaticity	C64175	C2H6O	46.1	929.5	326.402	1.12367
5	2-Propanol	Alcohol, spicy	C67630	C3H8O	60.1	920.2	320.364	1.2172
6	3-Methyl butanal	Chocolate, fat	C590863	C5H10O	86.1	919.5	319.933	1.40653
7	2-Butanone	Fruity, camphor	C78933	C4H8O	72.1	902.8	310.444	1.24685
8	Dimethyl sulfide	Cabbage, sulfur, gasoline	C75183	C2H6S	62.1	732.7	235.029	0.96112
9	Methyl acetate (M)	Ethereal	C79209	C3H6O2	74.1	831.3	276.178	1.03031
10	Methyl acetate (D)	Ethereal	C79209	C3H6O2	74.1	829.6	275.423	1.19341
11	Tetrahydrofuran (M)	Ether	C109999	C4H8O	72.1	861.5	290.146	1.06491
12	Tetrahydrofuran (D)	Ether	C109999	C4H8O	72.1	863.9	291.278	1.23013
13	Butyraldehyde (M)	Pungent, fruity, green leaf	C123728	C4H8O	72.1	874.5	296.375	1.11433
14	Butyraldehyde (D)	Pungent, fruity, green leaf	C123728	C4H8O	72.1	875.2	296.752	1.28379
15	Acetic acid ethyl ester	Fresh, fruity, sweet, grassy	C141786	C4H8O2	88.1	883.4	300.716	1.33886
16	2-Methyl-2-propanol	Camphor	C75650	C4H10O	74.1	919	319.592	1.32474
17	2,3-Butanedione	Butter, popcorn, sweet taste, sour rice	C431038	C4H6O2	86.1	984.3	364.218	1.17336
18	Ethyl 2-methylpropanoate	Sweet, fruity, alcoholic, rummy	C97621	C6H12O2	116.2	982.2	362.716	1.20359
19	n-Pentanal	Green grassy, faint banana, pungent	C110623	C5H10O	86.1	988.1	367.033	1.42479
20	3-Pentanone	Ethereal	C96220	C5H10O	86.1	986.5	365.8	1.36013
21	2-Pentanone	Acetone, fresh, sweet fruity, wine	C107879	C5H10O	86.1	988.1	367.033	1.39303
22	1-Propanol (M)	Alcohol, pungent	C71238	C3H8O	60.1	1040.8	418.528	1.10943
23	1-Propanol (D)	Alcohol, pungent	C71238	C3H8O	60.1	1039.9	417.603	1.2501
24	1-Hexanal	Fresh, green, fat, fruity	C66251	C6H12O	100.2	1092.8	478.668	1.26189
25	2-Methyl-1-propanol	Fresh, alcoholic, leather	C78831	C4H10O	74.1	1103.3	493.262	1.16829
26	(E)-2-Pentenal (M)	Potato, peas	C1576870	C5H8O	84.1	1140.5	553.195	1.10957
27	(E)-2-Pentenal (D)	Potato, peas	C1576870	C5H8O	84.1	1140.2	552.635	1.36083
28	Butanol (M)	Wine	C71363	C4H10O	74.1	1152.5	573.916	1.18159
29	Butanol (D)	Wine	C71363	C4H10O	74.1	1152.8	574.476	1.37801
30	1-Penten-3-ol	Ethereal, green, tropical fruity	C616251	C5H10O	86.1	1169.3	604.437	0.94097
31	Heptaldehyde	Fresh, aldehyde, fatty, green herbs	C111717	C7H14O	114.2	1190.3	644.759	1.33464
32	(E)-2-hexenal (M)	Green, banana, fat	C6728263	C6H10O	98.1	1227.8	694.369	1.18149
33	(E)-2-hexenal (D)	Green, banana, fat	C6728263	C6H10O	98.1	1227.8	694.369	1.52286
34	(Z)-4-heptenal	Grass, oil	C6728310	C7H12O	112.2	1250.1	724.714	1.14805
35	3-Methyl-3-buten-1-ol	Sweet, fruity	C763326	C5H10O	86.1	1257.7	735.503	1.17452
36	1-Pentanol	Balsamic	C71410	C5H12O	88.1	1259.7	738.201	1.25394
37	Ethyl 2-hydroxypropanoate (M)	Fruity	C97643	C5H10O3	118.1	1354.5	889.926	1.1383
38	Ethyl 2-hydroxypropanoate (D)	Fruity	C97643	C5H10O3	118.1	1352.6	886.577	1.53953
39	(Z)-2-Penten-1-ol	Green, plastic, rubber	C1576950	C5H10O	86.1	1334.7	855.354	0.94922
40	1-Hydroxy-2-propanone	Pungent, caramel, fresh	C116096	C3H6O2	74.1	1308.7	812.046	1.23504
41	3-Methylbutan-1-ol	Whiskey, banana, fruity	C123513	C5H12O	88.1	1217.1	680.138	1.24626
42	1-octanal	Aldehyde, waxy, citrus, orange, fruity, fatty	C124130	C8H16O	128.2	1294.9	789.931	1.40501

As shown in Figure 2B, the volatile flavor components of shrimp paste were found to consist of 34% alcohols, 31% aldehydes, 14% ketones, 14% esters, 5% furans, and 2% ethers. These components collectively form the distinctive flavor profile of shrimp paste. Among the volatile constituents of shrimp paste, aldehydes and alcohols are the predominant classes. Aldehydes, which primarily originate from the oxidation of fatty acids and degradation of amino acids, exhibit strong fruity, floral, or fatty aromatic profiles (19) and playing a crucial role in flavor formation. Alcohol compounds are predominantly short-chain aromatic alcohols, whose aromas are characterized by mild fruity, grassy, and fatty notes, synergizing with aldehydes, ketones, and other compounds. Ketone compounds are largely generated through β-oxidation of fatty acids, imparting sweetness and a creamy aroma to shrimp paste (20).

#### 3.1.3 Fingerprint spectrum comparison analysis

For the purpose of clarifying the impacts of maturation methods on the volatile flavor profile of shrimp paste, GC-IMS fingerprinting was utilized to analyze the compositional characteristics of the target compounds. The volatile component fingerprint spectra of shrimp paste processed by different maturation methods are shown in Figure 2E Significant differences exist in the volatile component patterns between the steamed group (ZK20, ZK25, ZCA, ZCC) and the stir-fried group (CK20, CK25, CCA, CCC). Volatile substance variations were smaller within the steamed group samples, while the CCC sample in the stir-fried group exhibited the greatest variation. Region B of the steamed group (e.g., 2-methyl-1-propanol, butanal, propanal) exhibited significantly higher concentrations of aldehydes and alcohols. This aligns with the retention of primary reaction products such as fatty acid oxidation and amino acid degradation under mild moist-heat conditions (21), which contribute to the harmonious savory and fresh flavor profile of shrimp paste. In contrast, stir-fried samples exhibited greater compositional heterogeneity. CCC samples showed significant enrichment of esters, ketones, and heterocyclic compounds in Region A, likely resulting from accelerated secondary reactions like Maillard cascade reactions and lipid oxidation rearrangements under high-temperature dry-heat conditions (22). While this process yields more complex flavors, it also increases the risk of batch-to-batch variability and off-flavors derived from over-reaction. The steaming process preserves key aldehyde and ketone flavor compounds to maintain sensory uniformity, while the stir-frying process promotes the formation of esters and alcohols (23). This clearly demonstrates that different cooking methods significantly influence the volatile flavor compounds in shrimp paste samples.

The key volatile organic compounds that drive aroma alterations in VIP shrimp paste subjected to different processing techniques were identified. A VIP value > 1 implies that the corresponding variable serves as a core component of the discriminant model, where higher VIP values reflect more pronounced differences among variables (24). By applying the screening criteria of *p* < 0.05 and VIP > 1, six core volatile aroma components with differential characteristics were determined (Figure 2D). These components include one aldehyde (Propanal), three alcohols [Ethanol, 1-Penten-3-ol, and (Z)-2-Penten-1-o]), one ketone (2-propanone), and another compound (Tetrahydrofuran). Propanal is produced from alanine during thermal degradation and exhibits intense fruity and nutty aromas. Propanal exhibits a higher flavor contribution value in the steamed group compared to the stir-fried group. Propanal, which imparts fruity and green leafy aromas, has also been identified during fish sauce fermentation (25). Ethanol acts as a characteristic flavor compound in boiled beef and contributes a meaty aroma to shrimp paste, as demonstrated by Yi et al. (26). 2-Propanone is primarily generated through the β-oxidation of saturated fatty acids, and this compound has also been detected in Guizhou white sour soup, where it significantly modulates shrimp paste flavor by balancing aquatic meatiness and fruity notes (18). Additionally, (Z)-2-penten-1-ol—with an odor reminiscent of cabbage—was found at elevated levels in virgin olive oils (27), and our results show it contributed more prominently to the flavor profile of the steamed group compared to the stir-fried group. Tetrahydrofuran is another volatile flavor compound characteristic of post-cooking processes. It has been detected in various seafood products, such as hot-air-dried oysters (28).

#### 3.1.4 Volatile component cluster analysis

The results of PCA performed on volatile components from the eight shrimp paste samples are illustrated in Figure 2C. PC1 contributed 49.0%, PC2 contributed 21.3%, and the third principal component (PC3) contributed 12.0%, with a cumulative contribution of 82.3%. This indicates that the model effectively reflects flavor differences among the samples. The CCC sample was positioned farther from other samples in the PCA plot, indicating its flavor characteristics differed significantly from the others. Samples CCA, CK20, and CK25 from the stir-frying group clustered closely but were clearly separated from the steaming group samples. The four steaming group samples showed highly concentrated distribution, suggesting good flavor consistency within this group. This result aligns with fingerprint analysis, further confirming that different maturation methods are the primary factors causing flavor differences in shrimp paste.

### 3.2 Electronic nose analysis results

**FIGURE 3:**
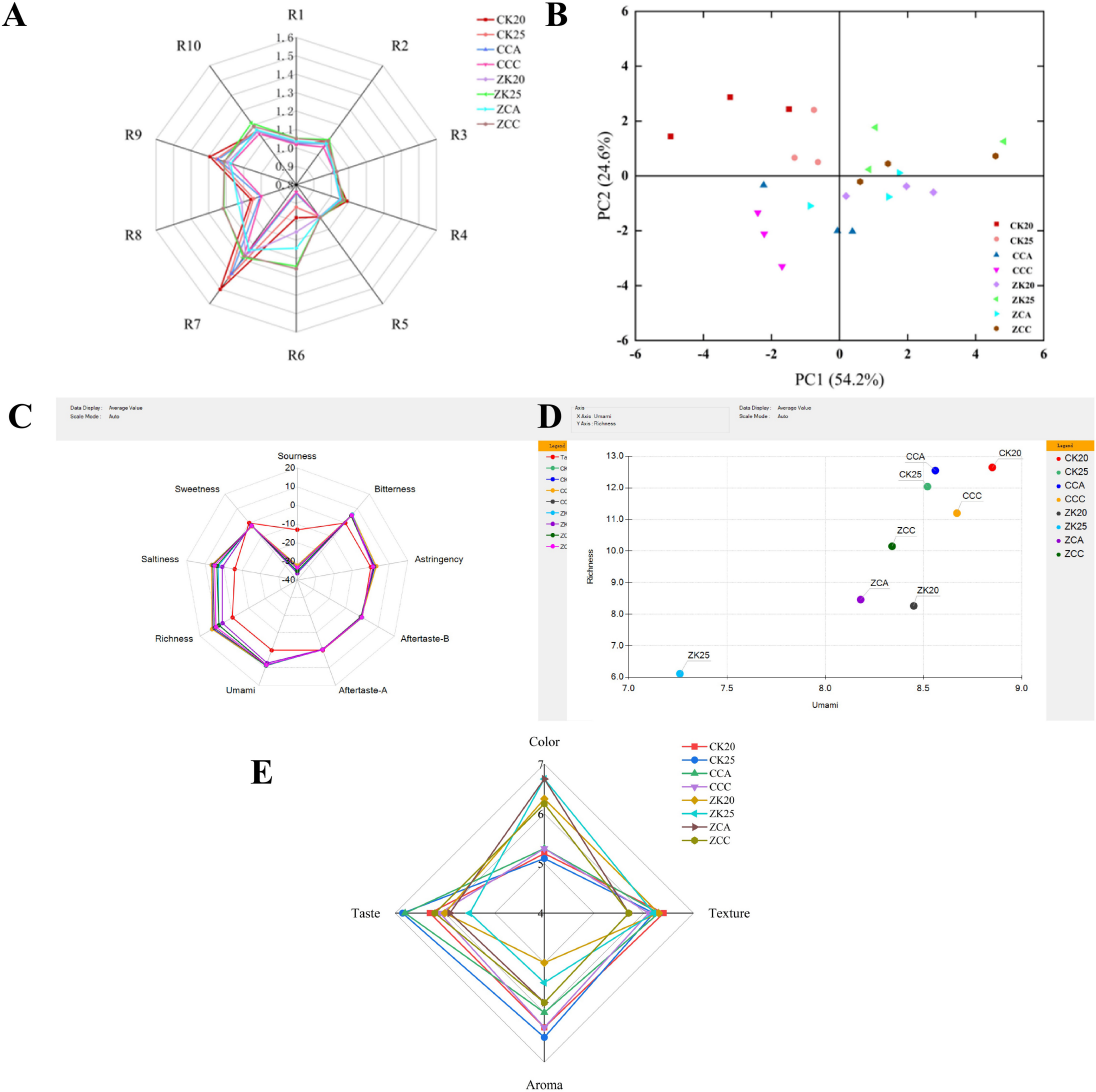
Radar diagram **(A)** and PCA diagram **(B)** of electronic nose of shrimp paste cooked in different ways; Cooked shrimp paste in different ways, electronic tongue radar chart **(C)**; scatter plot **(D)** and effect of different heat treatments on the sensory score of cook shrimp paste in different ways **(E)**.

The radar charts and PCA analysis results for the electronic nose sensors are shown in Figures 3A,B. The differences in volatile flavor profiles among the eight shrimp paste samples were primarily concentrated on sensors R6 (sensitive to methyl compounds), R7 (sensitive to inorganic sulfides), R8 (sensitive to alcohols, aldehydes, and ketones), and R9 (sensitive to organic sulfur compounds) (29). Responses on sensors R3 and R5 were nearly identical, indicating minimal differences in ammonia and short-chain alkane content among the shrimp pastes. The R7 sensor exhibited the highest response values, indicating that inorganic sulfides constitute one of the primary volatile components in shrimp paste. Furthermore, the response values for the stir-fried group (CK20, CK25, CCA) were higher than those for the steamed group, suggesting that the stir-frying process promotes the formation of inorganic sulfides (30). Sensors R6 and R8 exhibited higher response values in the steamed group than in the stir-fried group, indicating richer levels of methyl compounds and alcohol-aldehyde-ketone substances in the steamed group. In PCA analysis, the first principal component contributed 54.2%, with the second principal component contributing 24.6%, totaling 78.8% cumulative contribution. Samples CK20, CK25, and CCC from the stir-frying group were effectively distinguishable from other samples, whereas samples from the steaming group could not be clearly differentiated. This aligns with the PCA results from GC-IMS, demonstrating that the electronic nose can rapidly and accurately distinguish shrimp paste samples processed by different maturation methods.

### 3.3 Electronic tongue analysis results

Utilizing an artificial lipid membrane sensing technology which imitates the functional mechanism of taste bud cells, the electronic tongue allows the corresponding taste sensor to detect flavors in a manner close to human sensory experience. By monitoring potential fluctuations on the surface of the artificial lipid membrane, the sensor accomplishes taste detection and conducts evaluations of various flavor types (31). The radar chart of the electronic tongue is shown in Figures 3C,D. “Tasteless” represents the taste threshold point, with the threshold for sourness at -13 and for saltiness at -6. A sample’s taste value below the tasteless threshold denotes the lack of the corresponding taste, whereas a value higher than the threshold indicates that the taste is present (32). Richness is defined as the umami aftertaste, which reflects the duration of umami sensation in the sample and is also referred to as umami persistence. In contrast, bitter aftertaste denotes the residual intensity of bitterness, while astringent aftertaste indicates the residual intensity of astringency (33). For both maturation methods, shrimp paste samples exhibited acidity, bitterness/astringency aftertaste, and sweetness below the Tasteless point, while umami, richness, and saltiness were significantly above the Tasteless point. This indicates that umami, richness, and saltiness are the primary taste characteristics of shrimp paste (34). The umami response values of the stir-fried group were slightly higher than those of the steamed group, indicating that the stir-frying process more effectively enhances the umami of shrimp paste. Richness reflects the persistence of umami. The richness of shrimp paste under both maturation methods showed no significant difference but remained at a high level, suggesting that Antarctic krill paste possesses good umami persistence (35). Saltiness primarily originates from the salt content in shrimp paste. The saltiness response values among different samples showed minimal variation, indicating that salt concentration and the amount of aged shrimp paste added have limited effects on the saltiness of shrimp paste.

### 3.4 Free amino acid analysis results

During shrimp paste fermentation, endogenous proteases and microbial proteases degrade proteins into free amino acids (36). Accordingly, the types and concentrations of these amino acids exert a pivotal role in the formation of flavor profiles. As important taste-active compounds and flavor precursors, free amino acids contribute to the development of umami, saltiness, sweetness, astringency, and ester-derived aromatic notes in food products (37). Free amino acid analysis results are shown in Table 3. Shrimp paste is highly popular due to its distinctive salty-umami flavor, with amino acids contributing to umami including aspartic acid and glutamic acid. Among these, the glutamic acid content in stir-fried shrimp paste was slightly higher than that in steamed shrimp paste. The sweetness-contributing amino acids in matured shrimp paste were higher, including glycine, serine, proline, and lysine. Proline and glycine contributed most significantly to the sweetness of several shrimp pastes, with the stir-fried group exhibiting higher sweetness-contributing amino acid content than the steamed group. Alanine imparts umami and sweetness to shrimp paste, exhibiting relatively high levels and significant contribution across the tested samples (38). The content of bitter-tasting amino acids was higher in the stir-fried group than in the steamed group. Previous studies have shown that bitter-tasting amino acids can participate in the Maillard reaction and Strecker aldehyde degradation, thereby generating numerous flavor compounds (39). Common bitter amino acids include phenylalanine, histidine, arginine, leucine, isoleucine, and valine. Although bitterness is generally perceived as an unpleasant taste, bitter amino acids can contribute to the umami and richness of a system (40). Glutamic acid and phenylalanine are present in higher concentrations in several shrimp pastes, with glutamic acid being the most abundant bitter amino acid. The bitterness of glutamic acid can be masked by NaCl and glutamic acid, thereby enhancing its sweetness (41), suggesting this amino acid may also contribute to sweetness perception. The higher FAA content in the stir-frying group is closely associated with heat-induced protein degradation, which is modulated by the unique heat transfer characteristics of the stir-frying process. Specifically, stir-frying uses oil as a heat transfer medium, enabling shrimp paste to be heated rapidly and locally, thereby inducing moderate protein denaturation and hydrolysis without causing excessive aggregation (42).

**TABLE 3:** The contents of FAAs in cooking shrimp paste in different ways.

Compounds	Mass concentration/(μg/mL)							
	CK20	CK25	CCA	CCC	ZK20	ZK25	ZCA	ZCC
Aspartic acid	150.90 ± 2.20^a^	139.90 ± 1.05^b^	137.86 ± 3.04^b^	94.74 ± 0.67^e^	113.86 ± 2.25^c^	107.29 ± 5.34^d^	94.35 ± 1.30^e^	109.98 ± 0.75^cd^
Threonine	397.41 ± 1.27^b^	443.62 ± 1.83^a^	442.74 ± 1.51^a^	321.92 ± 2.63^f^	341.75 ± 0.86^e^	355.16 ± 0.27^d^	302.18 ± 0.86^g^	361.01 ± 1.97^c^
Serine	411.80 ± 1.61^c^	473.34 ± 1.70^b^	484.07 ± 1.03^a^	362.26 ± 1.48^e^	335.53 ± 1.97^f^	359.39 ± 9.52^e^	308.60 ± 1.14^g^	372.41 ± 4.00^d^
Glutamic acid	352.81 ± 1.26^b^	465.73 ± 1.52^a^	456.17 ± 3.66^a^	337.46 ± 1.75^bc^	303.82 ± 2.65^d^	346.50 ± 29.41^bc^	264.40 ± 4.98^e^	329.25 ± 4.47^c^
Proline	3732.71 ± 8.23^ab^	4412.29 ± 8.35^a^	4409.29 ± 11.81^a^	3464.81 ± 2.89^b^	3140.05 ± 31.78^e^	3286.88 ± 57.61^bc^	2569.07 ± 23.91^c^	3209.38 ± 14.19^bc^
Glycine	3164.15 ± 2.45^c^	3733.97 ± 9.79^a^	3706.61 ± 7.69^b^	2865.56 ± 2.39^d^	2592.20 ± 16.60^g^	2799.68 ± 20.97^e^	2213.25 ± 5.05^h^	2770.55 ± 9.17^f^
Alanine	1221.90 ± 4.99^c^	1385.00 ± 2.13^b^	1415.23 ± 2.23^a^	1073.21 ± 2.59^d^	1004.49 ± 8.06^f^	1045.86 ± 15.53^e^	852.38 ± 7.8^g^	1052.59 ± 7.03^e^
Valine	523.80 ± 2.15^b^	567.87 ± 1.18^a^	581.39 ± 3.02^a^	460.10 ± 2.11^c^	448.62 ± 13.52^c^	454.02 ± 24.60^c^	379.53 ± 10.39^d^	441.92 ± 10.39^c^
Methionine	297.12 ± 0.67^b^	325.06 ± 0.77^a^	328.34 ± 2.12^a^	272.87 ± 2.77^bc^	251.66 ± 31.09^c^	269.13 ± 23.03^bc^	214.67 ± 20.84^d^	250.27 ± 2.20^c^
Isoleucine	318.12 ± 2.12^ab^	323.27 ± 2.61^ab^	336.59 ± 2.99^a^	286.29 ± 3.49^bc^	257.83 ± 60.05^c^	268.18 ± 24.33^c^	207.39 ± 34.14^d^	249.20 ± 1.24^cd^
Leucine	576.91 ± 2.46^b^	622.17 ± 2.08^a^	637.05 ± 2.56^a^	483.56 ± 0.55^d^	493.33 ± 24.60^cd^	507.44 ± 19.55^c^	432.02 ± 16.76^e^	499.69 ± 0.95^cd^
Tyrosine	245.66 ± 2.00^b^	266.33 ± 1.60^a^	261.93 ± 2.40^a^	213.71 ± 1.37^d^	228.36 ± 6.89^c^	229.27 ± 18.20^c^	186.33 ± 5.70^e^	211.58 ± 1.58^d^
Phenylalanine	2322.96 ± 5.57^c^	2731.83 ± 7.24^a^	2679.81 ± 3.72^b^	2105.22 ± 2.42^d^	1935.78 ± 27.31^g^	2050.28 ± 58.50^e^	1619.11 ± 9.04^h^	1996.51 ± 5.51^f^
Lysine	614.38 ± 3.88^b^	673.34 ± 13.66^a^	660.73 ± 1.11^a^	494.25 ± 0.66^f^	519.08 ± 5.77^e^	540.82 ± 27.63^d^	495.05 ± 0.63^f^	581.86 ± 1.69^c^
Histidine	138.15 ± 11.05^b^	198.49 ± 40.26^b^	157.05 ± 1.07^b^	128.28 ± 0.95^b^	115.26 ± 12.69^b^	120.53 ± 17.16^b^	99.45 ± 8.44^a^	107.69 ± 5.84^b^
Arginine	3988.16 ± 0.85^c^	4894.89 ± 82.21^a^	4711.61 ± 2.79^b^	3730.02 ± 3.50^d^	3469.86 ± 16.39^f^	3757.55 ± 14.40^d^	2986.19 ± 2.69^g^	3655.12 ± 9.44^e^

Values are presented as mean ± standard deviation (*n=3*). Different lowercase superscript letters (a–f) indicate significant differences among treatments (*p* < 0.05, Duncan’s multiple range test).

Overall, the free amino acid content was higher in the stir-fried group, primarily comprising umami, sweet, and bitter amino acids. The stir-fried group exhibited higher free amino acid levels than the steamed group, indicating that stir-frying imparts a more intense flavor to the shrimp paste. Among the eight shrimp paste samples tested by the electronic tongue, umami and saltiness scores exceeded the neutral taste point. This result aligns with the free amino acid analysis, which showed higher concentrations of umami and sweetness-related amino acids in the stir-fried group compared to the steamed group.

### 3.5 Sensory evaluation results

The sensory evaluation radar chart Figure 3E indicates that sensory evaluation serves as an indicator reflecting shrimp paste quality. The sensory evaluations of different aged shrimp pastes are depicted as shown in the radar chart (43). Sensory evaluation was conducted across four dimensions: color, texture, aroma and taste. The figure reveals significant differences among the eight shrimp paste groups in color, aroma and taste, while no significant distinction was observed in texture. This indicates that the textural characteristics of stir-fried and steamed shrimp paste are largely comparable. The steamed group scored significantly higher in color than the stir-fried group. The oil-colored shrimp meat appeared bright, whereas continuous stirring during frying thoroughly mixed the shrimp meat with oil. This process, coupled with the Maillard reaction, resulted in a cloudier shrimp paste. In terms of flavor and aroma, CK25 received high marks, followed by CCA. As salt concentration decreased, overall evaluation scores gradually declined. Overall, the stir-fried group received higher overall ratings than the steamed group. This result, combined with electronic tongue and free amino acid analyses, suggests that the stir-frying process imparts a richer flavor to the shrimp paste, leading to higher overall evaluation scores compared to the steamed group.

## 4 Conclusion

This study systematically investigated the effects of two processing methods, steaming and stir-frying on the flavor, taste, and sensory quality of Antarctic krill paste using multiple analytical techniques. The following conclusions were drawn: A total of 42 volatile compounds were identified using GC-IMS technology, with aldehydes, ketones, and alcohols being the primary substances influencing the paste’s flavor. The steamed shrimp paste exhibited greater diversity in volatile flavor compounds and higher consistency, while the stir-fried paste showed greater flavor variation with elevated levels of certain esters and alcohols. The electronic nose effectively distinguished shrimp paste samples processed by different methods. The stir-fried group contained higher levels of inorganic sulfides and organic sulfur compounds than the steamed group, while the steamed group exhibited richer methyl compounds and alcohol-aldehyde-ketone substances. Electronic tongue and free amino acid analyses indicated that the stir-fried group exhibited higher umami response values and total free amino acids than the steamed group, with superior levels of umami, sweetness, and bitterness-related amino acids. Sensory evaluation revealed superior color in the steamed shrimp paste, while the stir-fried group scored higher in aroma, taste, and overall evaluation. The stir-fried sample with 25% salt content (CK25) exhibited the best overall quality. In summary, the stir-frying process is more suitable for the maturation treatment of Antarctic krill paste, and the 25% salt concentration formulation imparts superior flavor and taste qualities to the product. At the same time, the optimization of pilot-scale parameters for industrial production lines, quality control based on key biomarkers, and semi-industrial validation conducted in collaboration with seafood manufacturers will directly translate laboratory research findings into large-scale applications, providing practical technical guidance to enhance the products’ market competitiveness.

## Data Availability

The original contributions presented in the study are included in the article/Supplementary material, further inquiries can be directed to the corresponding author.
