# Supplementary material for: GC-IMS combined with sensory and chemical analyses to reveal flavor and taste differences of antarctic krill paste under different processing conditions
Source: Front Nutr. 2026 Apr 13;13:1805406. doi: 10.3389/fnut.2026.1805406 (PMC13110965; doi:10.3389/fnut.2026.1805406)
Supplement: Supplementary file 1 [file Table_1.docx]

**Supplementary Table 1** PEN3 Electronic Nose Sensor Performance Description.

| Sensor number | Sensor name | Performance description |
| --- | --- | --- |
| R1 | W1C | Sensitive to aromatic ingredients |
| R2 | W5S | High sensitivity, sensitive to nitrogen oxides |
| R3 | W3C | Sensitive to ammonia and aromatic components |
| R4 | W6S | Mainly selective for hydrogen |
| R5 | W5C | Sensitive to short-chain alkanes, aromatic components |
| R6 | W1S | Sensitive to short-chain alkanes such as methane |
| R7 | W1W | Sensitive to inorganic sulfides |
| R8 | W2S | Sensitive to alcohols, aldehydes, ketones, ethers, etc. |
| R9 | W2W | Sensitive to aromatic components and organic sulfides |
| R10 | W3S | Sensitive to long-chain alkanes |
